# Cases of Plica Polonica, in Men and Animals

**Published:** 1809-12

**Authors:** M. Roussille Chamseru


### *Cases of Plica Polonica, in Men and Animals.*
By M. Roussille Chamseru.*

(From the Journal de Medecine.)

Case I. A dog, 5 years old, of the middle size and fat, of a mixed breed, with long straight hair, had his left thigh broken by a club when 6 months old.

This animal was clumsy in his gait, dragging his hinder parts, and could not run fast; he seldom ventured to leave the premises which he watched, and lay almost constantly immersed in filth. Three years ago, during the autumnal months, he contracted on the buttocks a plica, with flat clots, two inches broad, and five or six in length, from which an unctuous matter issued. It was most conspicuous on the side of the thigh, and there were but few traces of it towards the tail, which was cut when a few days old. There is no plica on his back, shoulders, or head, although his hair is long, and his eyes are hid by it. It is the only dog in the town of Posen, in Poland, known to have the plica, and every person accounts for it in his own way. The most enlightened of the community, only regard it as the effects of the nastiness and lameness of the animal.

The mass of mankind, and the faculty, are of a contrary opinion; they concur in asserting, that there is a peculiar virus in the plica of men and animals. This idea has been long propagated, accompanied with superstitious prejudices, giving the character of an endemical disease to the plica of Poland. In my opinion, however, the disease is merely accidental.

Case II. — The master of this dog, a mechanic, 31 years old, of a robust, a nervous, but not muscular frame, and having a large family, has been ill for above two

---

* This paper is only the first of a controversy on plica; and, happily, the disease is not suspected of being indigenous in this country; we shall therefore only offer the leading points to our readers. Edit.

two years; he has been long bed-ridden; immersed in feather beds, and thus much exposed to violent perspiration. His hair of a dark brown, naturally thick, and which he was in the habit of combing over his shoulders, is clotted and matted with dirt, sweat, and vermin. This was the prelude to the plica, and it was easy to persuade him, that this *crisis* (for so it is called) of his malady, should be carefully watched. He has therefore suffered his hair, without either combing or cutting, to become matted and twisted into large clots, greasy and lousy, which spread in scales over each other, and present varieties of the plica. I cut off some as a specimen, as also some flocks from the right thigh of the above mentioned dog.

Popular opinion put great stress upon these two instances of plica, which came under my observation just as I was quitting Posen. The malady, they said, had passed from the dog to his master; which erroneous idea had given rise to a multitude of imaginary consequences, serving to propagate the old notion of the infectious and hereditary nature of the plica. It is however ascertained, that the animal did not infect any other, and that he had been the father of pups with long hair, some of which were alive, and quite healthy. The tradesman, who from prejudice was subjected to the plica, lodged with a numerous family on the ground floor of a small, healthy dwelling-house, situated in a well-aired street. Neither his wife, children, nor the other lodgers, felt at all this fictitious ailment.

Nothing is more common than to see the plica attack the masters of families, whilst the women, who attend somewhat to cleanliness, and the children, as long as they are properly combed, and their hair kept short, are not affected.

In general, it is very rare amongst men, but is much more intense when it gets hold of the fine head of hair of a female, who, under the predominant prejudice, allows her plica to increase for several years.

It is generally the plica of females, some yards in length, which is preserved in the museums of the curious, and I have endeavoured to trace the real mechanism of these immense superfœtations of hair.

The plica of the horse is likewise the result of the grease and filth of his hair. If these animals are never dressed, but left with all their grease and vermin, whilst the skin excoriates in places where the hair is cut away, with dirtiness, these brutes will infallibly catch the pretended pretended virus of the plica. The polish peasants do not venture to clean, dress, and rub down their horses, for fear of disturbing the plica, and irritating that malignant spirit, whose power and influence they imagine to rival that of providence.

If however, a young *konia*, (the Polish word for a horse) is already subject to the plica, presents a fine front, and other signs of comliness, as soon as he is taken from the hut of the slave into the stable of the nobleman, he is forthwith dressed and kept clean. The plica then begins to yield, and the veterinary art serves here as a lesson to men when attacked with this disease, which the military police also every year enforces on the Polish militia and recruits.

If any of them happen to have the plica, their locks are cut off and shaved without scruple or danger.

These observations on the plica, said to be endemical in Poland, have convinced me of a very strange mistake, spread over a whole country, and supported in their schools. I called to mind the famous story of the *golden tooth.* The *plica* cannot, in my opinion, be classed amongst internal maladies.

It is a local disorder of the hair from its length, neglect of combing, the wearing of warm furred bonnets, and the effects produced by dirtiness, sweat, and vermin. The hair, when kept short, never contracts the plica, which never degenerates into eruptive sores; the skin or scalp is always sound, and covered with free hair, if cut an inch or two from the root. It is not even practicable to cause the plica to assume an external or chirurgical disease; it is a mechanical consequence of filth, and belongs to the province of the toilette, or of the hair-dresser to cure.

On my way through Capel, a few months ago, I visited the museum founded by the Landgrave of Hesse, Frederick II. On looking at the stuffed lion and lioness, I perceived that the former had a great number of tuftes of the *plica*, at the root of his mane and along the belly, in the kind of fringe of long hair which proceeds from each side, and loses itself under the thighs. I remarked this singularity to the keeper of the museum, who had never before observed it. He was well acquainted, however, with the appearances of the *plica*, and he immediately recognized them with great astonishment. The lion had lived thirteen years in the menagerie of Capel, and died 4 years ago. His cage was twelve feet square. The menagerie still contained another lion with his female, which was big with with young. The flooring of their cage being frequently cleaned, was constantly moist. The old lion, when alive, *(without having been in Poland)* had easily caught the *plica*, while the almost shorn hairs of the female scarcely gave a lodgement to it. The parts which had been most exposed to filth and moisture, were most clotted with the *plica*. This is the true mechanical and local cause of this plica. A similar cause may have similar effects on long haired animals, placed in similar situations of slavery and filth.

I was anxious to visit the living lions in the menageric, but want of time prevented me.

